# A predictive model of bowel resection for incarcerated inguinal hernia based on the systemic immune-inflammation index

**DOI:** 10.3389/fsurg.2022.990481

**Published:** 2022-09-23

**Authors:** Lei Chen, Lei Chen, Ying-ying Wang, Li-xiang Zhang, Xiao-gang Xia

**Affiliations:** ^1^ Department of General Surgery, Xiang’an Hospital of Xiamen University, Xiamen, China; ^2^ Department of General Surgery, The First Affiliated Hospital of Wenzhou Medical University, Wenzhou, China; ^3^ Department of Emergency, Xiang’an Hospital of Xiamen University, Xiamen, China; ^4^ Department of Neurology, Xiang’an Hospital of Xiamen University, Xiamen, China; ^5^ Department of Gastroenterology, The First Affiliated Hospital of Anhui Medical University, Hefei, China

**Keywords:** systemic immune-inflammation index(SII), incarcerated inguinal hernia, nomogram, bowel resection, strangulated inguinal hernia

## Abstract

**Background and Purpose:**

An inguinal hernia is a common surgical disease. Once incarcerated or strangulated, it may endanger the life of the patient. Therefore, it is essential to study the risk factors of incarcerated inguinal hernia (IIH) and strangulated inguinal hernia (SIH). One of the serious complications of IIH and SIH is intestinal necrosis, which occurs owing to blood supply disorder. The study explores the risk factors of intestinal resection and establishes a simple model to assess the incidence of intestinal resection to provide significant assistance and limited guidance for clinical work.

**Patients and Methods:**

Our research team collected and retrospectively analysed the clinical data of 338 patients with IIH who were hospitalized in the First Affiliated Hospital of Wenzhou Medical University between September 2008 and December 2016. According to the surgical plan, we divided the included cases into two groups, non-intestinal and intestinal resection groups, and the clinical case characteristics of these groups were statistically analysed.

**Results:**

Based on multivariable logistic regression analysis, we found that increased risk of bowel resection was highly correlated among the elderly (≥70 years), and for people with high temperature (≥37.3°C), high systemic immune-inflammation index(SII) values (≥1230.13), presence of bowel obstruction, and signs of peritonitis. Further, we processed the five independent risk factors using special software to obtain a simple model called a nomogram. To verify the nomogram’s accuracy and predictive ability, we calculate the C-index: 0.806 and use the calibration curve to evaluate its stability and predictive performance. We constructed the ROC curve nomogram and other sub-variables, and calculated the area under the curve (AUC) corresponding to the nomogram (AUC = 0.808, 95% CI = 0.762 to 0.848), SII (AUC = 0.752, 95% CI = 0.703 to 0.797), age (AUC = 0.641, 95% CI = 0.587 to 0.692), temperature (AUC = 0.579, 95% CI = 0.524 to 0.632), bowel obstruction (AUC = 0.685, 95% CI = 0.633 to 0.734), and signs of peritonitis (AUC = 0.580, 95% CI = 0.525 to 0.633).

**Conclusion:**

It can be said that we found for the first time that clinical variables such as SII are independent risk factors for enterectomy for IIH. The nomogram based on SII and other variables can accurately and easily predict the probability of IIH requiring bowel resection.

## Introduction

External abdominal hernias occurring in the groin area are collectively referred to as inguinal hernias, which are the most common type of hernia, causing bulging in the groin area (1, 2). Further, they can also lead to pain and bowel obstruction (1, 2). Incarcerated inguinal hernia (IIH) is a common acute abdominal disease, and most patients with IIH require emergency surgery (3). Failing to reset IIH effectively can rapidly make the necrotic hernia content due to severe blood supply disorder. This condition is called strangulated inguinal hernia (SIH). The most effective treatment for IIHs is timely surgical intervention, especially in the case of SIHs (3). Due to prolonged incarceration and avascular necrosis of hernia content, approximately 15% of the patients with SIH require bowel resection (4). Therefore, it is essential to assess the risk of bowel resection prior to the surgery of patients with IIH. Based on our knowledge, apart from obvious peritonitis, there are no clear clinical criteria to distinguish among different strangulations. The study explores the risk factors of intestinal resection and establishes a simple model to assess the incidence of intestinal resection to provide significant assistance and limited guidance for clinical work.

## Patients and methods

### Study population

Our research team collected and retrospectively analysed the clinical data of 410 patients with IIH who were hospitalized in the First Affiliated Hospital of Wenzhou Medical University between September 2008 and December 2016. Our inclusion criteria were as follows: (1). Attainment of complete clinical case information; (2). All the cases should be that of IIH; (3). All should have successfully undergone surgery; (4). During the operation, the hernia content should be present in the intestinal tract; (5). No other severe concomitant disease should exist in the patient. The following were excluded based on the inclusion criteria: 31 cases of incomplete information, 24 cases of non-intestinal incarceration patients (the content of the incarceration was omentum), and 17 patients with severe concomitant diseases, such as patients with respiratory infections. All laboratory tests were performed after admission and before antibiotics. Finally, we successfully screened 338 cases, including 265 (78.4%) non-intestinal and 73 (21.6%) intestinal resection cases, respectively. Our research was supported by the ethics committee and Institutional Review Board of our hospital, and all the patients signed informed consent before participating in the research.

### Data collection and analysis

According to the surgical plan, we divided the included cases into two groups, non-intestinal and intestinal resection groups, and the clinical case characteristics of these groups were statistically analysed. The observed clinical variables were as follows: gender, age (years), body temperature (°C), height, weight, duration of incarceration (hours), presence or absence of bowel obstruction, presence, or absence of peritonitis signs, and presence or absence of chronic disease. The laboratory data included the following: neutrophil count, lymphocyte count, platelet count, fibrinogen, prothrombin time (PT), and activated partial thromboplastin time (APTT). Based on the height and weight, the body mass index was calculated using the formula: weight/height (2) (kg/m^2^). The systemic immune-inflammation index (SII) was calculated with laboratory variables using the formula: platelet count × neutrophil count/lymphocyte count.

### Statistical analysis

Continuous variables were divided into two groups depending on the cut-off value obtained according to a receiver operating characteristic (ROC) curve and maximum Youden’s index. Numbers (%) were used to identify categorical variables. The Mann-Whitney U and Chi-square tests were used to distinguish between the variables in these groups. The multivariable logistic regression analysis was used to screen the independent risk factors of intestinal resection for IIH patients, and the odds ratio (OR) and 95% confidence interval were calculated. Based on the obtained independent risk factors, scientific, accurate, and simple nomogram was constructed. The concordance index (C-index) and the calibration curve were used to verify the model’s prediction accuracy and expressiveness. The ROC curve was used to compare the difference between the model and other risk factors. Statistical analyses and drawing were implemented using IBM SPSS 21.0 (SPSS Inc, Armonk, NY) and R software (a language and environment for statistical computing. R Foundation for Statistical Computing, Vienna, Austria. URL https://www.R-project.org/). *P *< 0.05 was considered to be statistically significant.

## Results

### Patients’ characteristics

Among the 338 cases of IIH, 206 (77.7%) and 47 (21.6%) patients were male in the non-intestinal and intestinal resection groups. The median age of the patients was 70 years. A total of 73 patients were in the bowel resection group, accounting for 21.6% of the total cases. Further, the non-intestinal resection group accounted for 78.4% of the patients. The results of the univariate analysis of clinicopathological characteristics in our research group are listed in Table 1.

**Table 1:** Univariate analysis of clinical/laboratory parameters and incarcerated groin hernia patients with or without bowel resection.

Variables	No bowel resection	Bowel resection	*P* value
	*n* = 265 (78.4)	*n* = 73 (21.6)	
Gender			0.021*
Male	206 (77.7)	47 (64.4)	
Female	59 (22.3)	26 (35.6)	
Age (years)			0.002*
<70	127 (47.9)	20 (27.4)	
≥70	138 (52.1)	53 (72.6)	
Temperature (°C)			0.017*
<37.3	222 (83.8)	52 (71.2)	
≥37.3	43 (16.2)	21 (28.8)	
BMI (kg/m^2^)			0.115
<21.94	162 (61.1)	52 (71.2)	
≥21.94	103 (38.9)	21 (28.8)	
PT (S)			0.061
<13.5	131 (49.4)	27 (37)	
≥13.5	134 (50.6)	46 (63)	
Fibrinogen (g/l)			0.030*
<3.94	140 (52.8)	28 (38.4)	
≥3.94	125 (47.2)	45 (61.6)	
APTT (S)			0.769
<36.1	114 (43)	30 (41.1)	
≥36.1	151 (57)	43 (58.9)	
SII			0.000*
<1230.13	155 (58.5)	14 (19.2)	
≥1230.13	110 (41.5)	59 (80.8)	
Duration of incarceration (hours)			0.007*
<24	116 (43.8)	19 (26)	
≥24	149 (56.2)	54 (74)	
Bowel obstruction			0.000*
Presence	138 (52.1)	11 (15.1)	
Absence	127 (47.9)	62 (84.9)	
Signs of peritonitis			0.000*
Absence	253 (95.5)	58 (79.5)	
Presence	12 (4.5)	15 (20.5)	
With chronic disease			0.516
Absence	160 (60.4)	41 (56.2)	
Presence	105 (39.6)	32 (43.8)	

Abbreviation: BMI, body mass index; PT, prothrombin time; APTT, activated partial thromboplastin time; PLR, platelets -to-lymphocyte ratio; NLR, neutrophil-to-lymphocyte ratio; SII, systemic immune-inflammation index.

Notes: *P < 0.05.

### Risk factors associated with bowel resection of IIH patients

Based on multivariable logistic regression analysis, we obtained five independent risk factors for bowel resection after surgery for IIH, which were age, temperature, SII, bowel obstruction, and signs of peritonitis. We found that increased risk of bowel resection was highly correlated among the elderly (≥70 years), and for people with high temperature (≥37.3°C), high SII values (≥1230.13), presence of bowel obstruction, and signs of peritonitis (Table 2).

**Table 2:** Multivariable analysis of clinical/laboratory parameters and incarcerated groin hernia patients with or without bowel resection.

Variables	*P* value	OR	95%CI
Age (<70, ≥70 years)	0.038*	2.039	1.039–4.002
Temperature (<37.3, ≥37.3°C)	0.041*	2.153	1.033–4.487
SII (<1230.13, ≥1230.13)	0.000*	4.387	2.183–8.816
Bowel obstruction (absence, presence)	0.001*	3.498	1.627–7.518
Signs of peritonitis (absence, presence)	0.005*	3.727	1.492–9.312

Abbreviation: SII, systemic immune-inflammation index; OR, odds ratio; CI, confidence interval.

Notes: *P < 0.05.

### Nomogram for bowel resection of IIH

**Figure 1:**
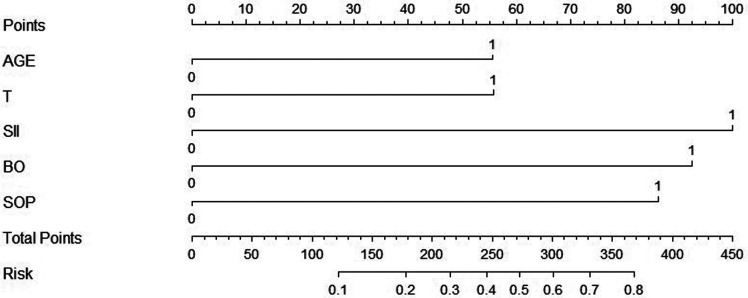
Nomogram for predicting the risk of enterectomy for incarcerated groin hernia. Abbreviation: AGE, age (0 means <70, 1 means ≥70 years); T, temperature (0 means <37.3, 1 means ≥37.3°C); SII, systemic immune-inflammation index (0 means <1230.13, 1 means ≥1230.13); BO- Bowel obstruction (0 means absence, 1 means presence); SOP- Signs of peritonitis (0 means absence, 1 means presence).

**Figure 2:**
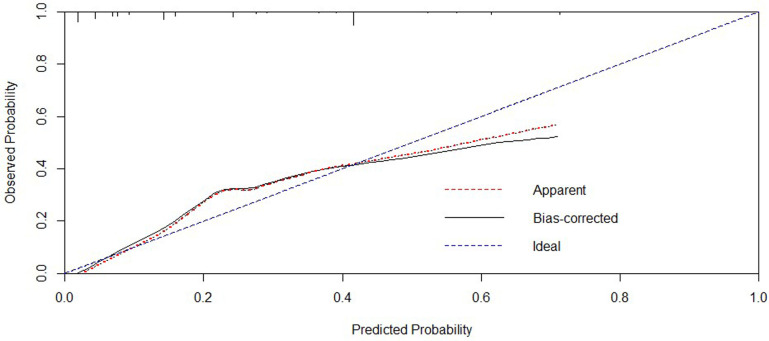
Calibration curve of the nomogram.

**Figure 3:**
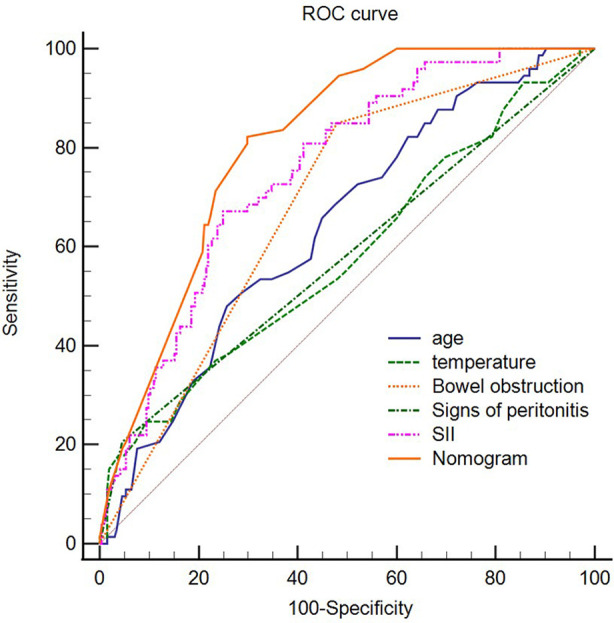
ROC curve of the nomogram, SII,temperature, bowel obstruction, signs of peritonitis. Abbreviation: SII, systemic immune-inflammation index; ROC, receiver operating characteristic.

Further, we processed the five independent risk factors using special software to obtain a simple model called a nomogram (Figure 1). Each sub-variable was observed to have been assigned a certain score. These scores were added to obtain the total score and determine the corresponding point on the total score scale. A vertical line was drawn in the downward direction from this point, which enabled an easy estimation of bowel resection risk probability. To verify the model’s accuracy, we calculated the C-index: 0.806, wherein a larger value indicated higher reliability of the model. The calibration curve was used to verify the performance of the model (Figure 2). Further, to confirm the predictive power of the model, we constructed the ROC curve nomogram and other sub-variables, and calculated the area under the curve (AUC) corresponding to the nomogram (AUC = 0.808, 95% CI = 0.762 to 0.848), SII (AUC = 0.752, 95% CI = 0.703 to 0.797), age (AUC = 0.641, 95% CI = 0.587 to 0.692), temperature (AUC = 0.579, 95% CI = 0.524 to 0.632), bowel obstruction (AUC = 0.685, 95% CI = 0.633 to 0.734), and signs of peritonitis (AUC = 0.580, 95% CI = 0.525 to 0.633) (Figure 3). Table 3 lists each variable’s exact boundary values in the nomogram and exact probability values of bowel resection.

**Table 3:** Nomogram scoring system.

Age (years)	Points	Temperature (°C)	Points	SII	Points	Bowel obstruction	Points	Signs of peritonitis	Points
<70	0	<37.3	0	<1230.13	0	without	0	no	0
≥70	56	≥37.3	56	≥1230.13	100	with	92	yes	86
Total points					Risk				
122					0.1				
178					0.2				
215					0.3				
245					0.4				
273					0.5				
301					0.6				
331					0.7				
368					0.8				

Abbreviation: SII, systemic immune-inflammation index.

## Discussion

An inguinal hernia is a common surgical disease. Once incarcerated or strangulated, it may endanger the life of the patient. Therefore, it is essential to study the risk factors of IIH and SIH. One of the serious complications of IIH and SIH is intestinal necrosis, which occurs owing to blood supply disorder.

In recent years, studies on the risk factors of IIH requiring bowel resection have been reported successively. Alvarez et al. reported that approximately 12.9% of 70 patients with inguinal hernia require bowel resection (5). Xie et al. found that the neutrophil-to-lymphocyte ratio (NLR) has clinical significance in predicting the severity of IIHs (6). Similarly, Zhou et al. believed that NLR is significant in diagnosing adult SIH (3). Compared to the previous method of judging whether an IIH has strangulation based on clinical manifestations and signs, NLR is an objective biological indicator calculated using laboratory data. Based on this, we concluded that the SII index is also an infectious index enabling us to understand if SII is inevitable based on the severity of IIH. Our team retrospectively analysed the clinicopathological data of 338 patients with IIH undergoing emergency surgery at our hospital-based on this conjecture. As expected, SII and other clinical indicators are closely related to the intestinal resection rate of IIH. Based on the literature, we found that SII has not been studied and reported by scholars as a new biological indicator for judging the severity of IIH. Although SII is closely related to IIH resection, wherein the probability of intestinal resection in patients with high levels of SII increases, the specific mechanism is still unclear. Based on previous reports, it is noted that scholars found SII to be closely related to the prognosis of tongue cancer (7), non-small cell lung cancer (8), colorectal cancer (9), intrahepatic cholangiocarcinoma (10), esophageal squamous cell carcinoma (11), and anal cancer (12). SII is calculated from the ratio of neutrophils, platelets, and lymphocytes. Nathan et al. confirmed that neutrophils are human immune cells, and as an indicator of inflammation, they can promote the formation and development of tumors. Further, an increase in neutrophils can inhibit lymphocyte production, which is a form of inflammation (13). Jenne et al. believed that platelets are effective immune modulators and effectors in the human body, which can identify, isolate, and kill pathogens and can enhance phagocytosis and the destroying ability of white blood cells (14). When IIH causes peritonitis, an inflammatory response is triggered in the body. This could be the reason why the higher the SII value calculated from the inflammation index is, the severe the inflammatory response in the body is, followed by a greater risk of bowel resection. The relevant mechanism is yet to be further studied by scholars.

The risk of bowel resection after strangulation of an incarcerated hernia is not just significantly related to SII but also closely related to the patient’s age, body temperature, intestinal obstruction, and signs of peritonitis. The risk of intestinal resection in patients over 70 years with an incarcerated hernia is significantly higher than that in patients who are less than 70 years old, which could be related to the decline in older patients’ immunity and physical functionality. Our study also found that intestinal obstruction is one of the independent risk factors for IIH undergoing enterectomy, consistent with previous studies. However, the specific mechanism is still not clear and may be related to intestinal necrosis caused by incarceration for an extended time. In addition, the presence of peritonitis significantly increases the risk of bowel resection. In addition, the presence of peritonitis also greatly increases the risk of bowel resection, which may be related to the inflammation caused by intestinal necrosis or intestinal perforation that stimulates the peritoneum (15).

After analysing the risk factors of enterectomy for IIH, we further developed a simple model called the nomogram to predict the intestinal resection rate intuitively and accurately. In recent years, nomograms have been widely used to predict prognosis or complications of various diseases, such as liver cancer (16), stomach cancer (17), rectal cancer (18), and small cell lung cancer (19). The nomogram graphically represents each predictor variable’s influence on the outcome, which enables the readers to have a specific explanation for the variable’s influence (20). To the best of our knowledge, our research team is the first to construct a nomogram to predict the rate of bowel resection for IIHs based on five clinicopathological variables, including SII. To verify the nomogram’s accuracy and predictive ability, we calculate the C-index: 0.806 and use the calibration curve to evaluate its stability and predictive performance. Both the C-index and the calibration curve indicate that our nomogram is accurate and stable.

Our research still has a few drawbacks, which are as follows: (1). Our research data includes only one institution and one region, and, hence, lacks universality; (2). Owing to strict inclusion criteria, this study is only applicable to incarcerated groin hernia, and the content of the hernia is intestinal tubes; (3). Our sample size is small, and a large sample is needed to confirm our research results further.

In conclusion, it can be said that we found for the first time that clinical variables such as SII are independent risk factors for enterectomy for IIH. The nomogram based on SII and other variables can accurately and easily predict the probability of IIH requiring bowel resection.

## Data Availability

The original contributions presented in the study are included in the article/Supplementary Materials, further inquiries can be directed to the corresponding author/s.
